# Oral health professionals screening for undiagnosed type-2 diabetes and prediabetes: the iDENTify study

**DOI:** 10.1186/s12902-022-01100-9

**Published:** 2022-07-18

**Authors:** Rodrigo Mariño, Andre Priede, Michelle King, Geoffrey G. Adams, Maria Sicari, Mike Morgan

**Affiliations:** 1 Melbourne Dental School, The University of Melbourne, Melbourne, Australia; 2 University of Otago, Dunedin, New Zealand

**Keywords:** Oral Health, Type-2 Diabetes, Oral Health Professionals

## Abstract

**Background:**

As part of an evaluation of an oral healthcare practice-based model that identifies patients with prediabetes or type-2 diabetes, this study reports on the proportion of patients identified with clinically confirmed type-2 diabetes (T2D)/prediabetes and barriers of implementation of the model.

**Methodology:**

Urban and rural oral healthcare practices were invited to participate. Participating practices invited eligible patients to participate in the screening program using the Australian Type-2 Diabetes Risk Assessment Tool (AUSDRISK). Participants were categorised as low, intermediate, or high-risk for prediabetes/T2D. Patients in the intermediate or high-risk category were referred to their General Medical Practitioner (GP) for further investigation.

**Results:**

Fifty-one oral healthcare practices and 76 Oral Health Professionals (OHP) participated (60 Dentists, 8 Dental Hygienists, 8 Oral Health Therapists). 797 patients were screened; 102 were low-risk; 331 intermediate-risk; and 364 high-risk for T2D. Of the 695 participants in the intermediate or high-risk groups, 386 (55.5%) were referred to their GP for T2D assessment. Of them, 96 (25.0%) results were returned to OHPs. Of the returned results, six were (6.3%) diagnosed with pre-T2D.

**Conclusion:**

Patients found to have undiagnosed T2D/prediabetes (6.3%) were within the expected range reported in the literature. Findings indicate that identifying individuals at an elevated risk of having or developing T2D is effective, feasible and could be incorporated into oral healthcare settings. However, this integration may require additional OHPs training and education to ensure that patients at elevated risk of T2D are referred for further assessment.

## Background

According to the latest International Diabetes Federation figures, 387 million people (8.3% of adults) worldwide are living with diabetes, and this expected to increase to 592 million people by 2035 [1]. A further 316 million people with impaired glucose tolerance are at high risk of diabetes, and this is expected to increase to 471 million by 2035. In 2014, diabetes accounted for over 10 percent of healthcare expenditure worldwide (US$ 911 billion) [1].

Early diagnosis and treatment of this disease is critical to improving health outcomes. Effective treatment reduces the risk of strokes, deterioration of vision and kidney failure by more than a third, more than halves the risk of heart failure and reduces the number of amputations and foot ulcers [2]. Although there has been some success in treating and preventing diabetes, the burden of diabetes, in terms of prevalence, continues to grow due to a combination of unfavourable risks factors and an ageing population [3].

People at high risk of developing diabetes (“prediabetes”) are in a lengthy asymptomatic stage that precedes overt type-2 diabetes [4]. and the complications of diabetes may begin even before people develop diabetes. In order to reduce the diabetes burden, a comprehensive diabetes strategy must prevent the development of diabetes, detect those with undiagnosed diabetes earlier, and improve the care for those with diabetes to prevent complications.

Early intervention can be only achieved with early identification of those with prediabetes or undiagnosed diabetes. This can result in significant savings in healthcare costs. Investment in early detection and referral for treatment is likely to reduce the healthcare costs of type 2 diabetes to the Australian economy, including healthcare expenditure, the cost of carers and government subsidies which are currently estimated to be $14.6 billion. This is forecast to increase to $30 billion by 2025 [5].

Management of the burgeoning diabetes pandemic requires new and innovative solutions that incorporate screening programs to detect undiagnosed type 2 diabetes and provide better access to all components of the multi-disciplinary teams and treatments [2]. Comprehensive care for people with diabetes must be a team effort involving the patient and a range of health care professionals. Oral Health Professionals (OHP) (dentists, dental hygienists, dental therapists, and oral health therapists) have an important, but often unrecognised role, in this multi-professional team [6].

There is considerable evidence to suggest a bidirectional relationship between diabetes and periodontal (gum) disease [7–10]. Numerous cross-sectional and prospective cohort studies have also shown that individuals with diabetes have more widespread or severe periodontal disease than individuals without diabetes [8, 11–14]. Periodontal infection can adversely affect glycaemic control and increase the risk of diabetic complications in people with diabetes.

Opportunistic identification of patients at high risk of diabetes using simple screening methods (such as in the dental setting) has been suggested as a preferred strategy over population-based screening as they can more accurately target candidates for screening and can help connect patients with other health providers [15]. Australians generally have favourable dental visiting patterns, and studies show that patients and dentists agree the dental visit is an appropriate site for diabetes screening [16]. The dental setting provides an important, but currently unrealised, additional opportunity for identification of prediabetes/undiagnosed diabetes as part of a multi-professional team approach.

The use of OHP to identify prediabetes and undiagnosed type-2 diabetes (T2D) has been trialled in a number of countries including the United States [4, 17–19], Greece [20], Saudi Arabia [21], Italy [22], Denmark [23], and Nigeria [24]. Studies [17, 25] have also explored the feasibility of random plasma glucose levels for screening for prediabetes or previously undiagnosed diabetes in community dental practices. Whilst these trials show promising results [17, 26], HbA1c testing in the dental office may not provide a feasible diabetes screening option for a number of reasons: the HbA1c test is an invasive procedure, most general dental practitioners would not have HbA1c devices in their offices, and it is unlikely that OHPs would adopt HbA1c testing if they are unable to claim reimbursement. A study which looked at risk identification based solely on a risk score, found that nearly half of the participants were identified as been at moderate or high risk [26].

However, to date, there is little information as to how a private dental practice-based screening model would work in the Australian context. A clinical study was designed to developed and evaluated an innovative approach for identifying type-2 diabetes and prediabetes using by oral health professionals recruited from private rural and urban dental practices throughout the Australian state of Victoria. The development of pathways of care between OHPs and general medical practitioners would also have benefits for other aspects of oral healthcare. This would benefit the Australian community as earlier diagnosis and management of diabetes would reduce long-term complications and potentially reduce downstream demands on the health care system more generally.

## Methodology

With the approval of the Human Research Ethics Committee at the University of Melbourne, three distinct groups of participants were approached:

- Oral Health Professionals (OHP) self-identified an interest in participating through newsletter advertising by oral health networks, Dental Hygienists Association of Australia Ltd (DHAA), Australian Dental and Oral Health Therapists Association (ADOHTA) and The University of Melbourne Alumni. Registration of interest could also be made via the study website (https://identifydiabetes.org/). OHPs also had the opportunity to register their interest in participating at the end of their completion of an Attitudes and Opinions to T2D screening in dental settings survey [27].

The OHPs were provided with a recruitment pack containing a study information brochure (plain language statement), study consent form and information about T2D and oral health. Consenting OHPs implemented the iDENTify patient recruitment and screening protocol by asking their patients to participate in the study.

Potential patients of participating oral healthcare practices were provided with a participant pack that included the plain language statement, patient informed consent form, the Australian Diabetes Risk Assessment Tool (AUSDRISK) [28] and information about T2D and oral health. AUSDRISK identifies individuals at high risk of developing type 2 diabetes, and its score includes questions based on age, gender, ethnicity, family history of diabetes, history of high blood glucose, hypertension, smoking status, fruit and vegetable intake, physical activity and waist circumference. The AUSDRISK score relates to the probability of developing diabetes within the next 5 years [29].

The AUSDRISK was developed using data from Australia. Other risk assessment tools have been developed to predict the development of type 2 diabetes, but their validity and applicability to the Australian population is debatable [30].

Eligibility criteria include dentate men and women aged 35 years or older; of any racial or ethnic background; able to undergo a periodontal examination; having never been told by a physician that they have diabetes or prediabetes. Exclusion criteria included health reasons (i.e., diseases or neurologic diseases that would compromise the patient’s ability to participate in the study), attending as an emergency visit, having a history of, or currently having T2D and if female; being pregnant.

The AUSDRISK tool categories people as Low risk (≤5); Intermediate risk (6-11); or High risk (≥12) of type 2 diabetes [28]. Eligible participants who had and AUSDRISK score of 6 and over were considered to be at increased risk for having prediabetes or undiagnosed diabetes and were invited to participate in the study.

b) If the OHP’s assessment deemed their patient as at risk of prediabetes or T2D, the OHP provided a referral letter and information pack for the patient recommending that they speak to a General Medical Practitioner (GP), and an information pack to take to their GP. This would ensure the patient is engaged with a GP for their care, regardless of the final diagnosis The GP information pack included a form for the GP to provide an assessment of the patients T2D or prediabetes status back to OHP.

In Australia three laboratory tests may be used for diagnosing type 2 diabetes: HbA1c test; Fasting Blood Glucose (FBG); or an Oral Glucose Tolerance Test (OGTT). Commonly, HbA1c and FBG are used, and if type 2 diabetes is likely it is confirmed with a repeat test [31].

**Fig. 1:**
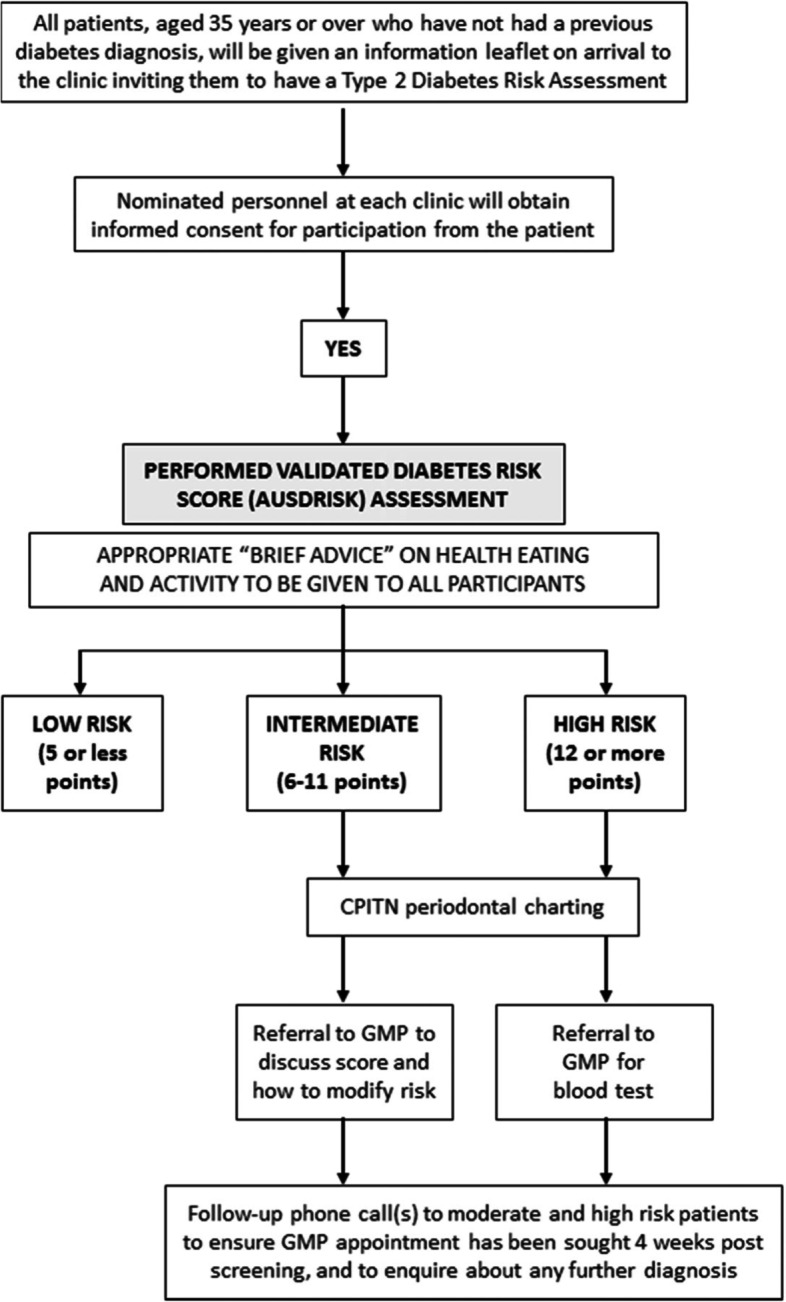
Patient progress flow chart

A flow chart outlining patient progress through the study is summarised in Fig. 1. The coordinator followed up with referred patients. Those who do not accept a referral were asked for the reason(s) behind the non-acceptance.

Tailored completion surveys were provided for OHPs and participating patients. Due to the increased pressures on General Practice in March–April 2020, following the emergence of COVID-19 in Australia, it was deemed an inappropriate time to send the surveys to GPs. These surveys measured acceptability of the screening program as well as a barriers and facilitators to program uptake. OHPs had the opportunity to provide a reason as to why the intermediate or high-risk participants were not referred.

Data collection commenced in September 2018 and was completed in March 2020. It was estimated that if 50 to 60 OHPs were involved in the study, they would recruit some 15 patients each, giving a total sample of approximately 700 to 900 patients. This sample size allowed the population proportion to be estimated with an absolute precision of ± 5%, assuming a clustered sampling design, that the underlying population proportion of people with either prediabetes or undiagnosed is 20% and an intra-class correlation of 0.05 [32]. This sample size is in accordance with previous studies [4, 18, 24].

Our primary outcome was the proportion of patients identified with clinically confirmed diabetes/prediabetes. Participating OHPs, patients and GPs were asked to complete a survey to measure self-reported acceptability of the screening program as well as a barriers and facilitators to program uptake.

Data manipulation and analyses were conducted using IBM SPSS Statistics (Version 23.0, IBM Corporation, Endicott, NY, USA). Continuous variables, reported as mean and standard deviations, and categorical variables, reported as counts (percentages), were compared between those identified as low, intermediate, and high risk and *p*-values lower than 0.05 were considered statistically significant.

## Results

A total of 51 Victorian oral healthcare practices agreed to participate. 76 OHPs (60 Dentist, 8 Dental Hygienists and 8 Oral Health Therapists) participated. Of the participating oral healthcare practices, 34 practices were in the metropolitan Melbourne area and 17 where in rural Victoria. The patient participant recruitment averaged 15.8 patients per practice (range: 1–31).

806 patients participated in the study. Of those, nine were excluded in the final analysis: one due to incomplete data, three because they did not satisfy the age criteria (i.e., 35 and older), and one participant withdrew their consent. Four participants advised their OHP they had been diagnosed with T2D or pre-T2D by their GP. Participants overall mean age was 56.7 (SD 12.6) years, ranging from 35 to 92 years and more participants were female (61.2%) (See Table 1).

**Table 1:** Demographic characteristics of patient participants

	**Participants**	**%**
**Location**		
Metro	574	72.0
Rural	225	28.0
**Sex**		
Female	488	61.2
Male	308	38.8
**Age Group**		
34–44 years	150	18.8
45–54	206	25.8
55–64	198	24.9
65–74	167	21.0
75 and more	76	9.5
**Total**	797	100.0

**Fig. 2:**
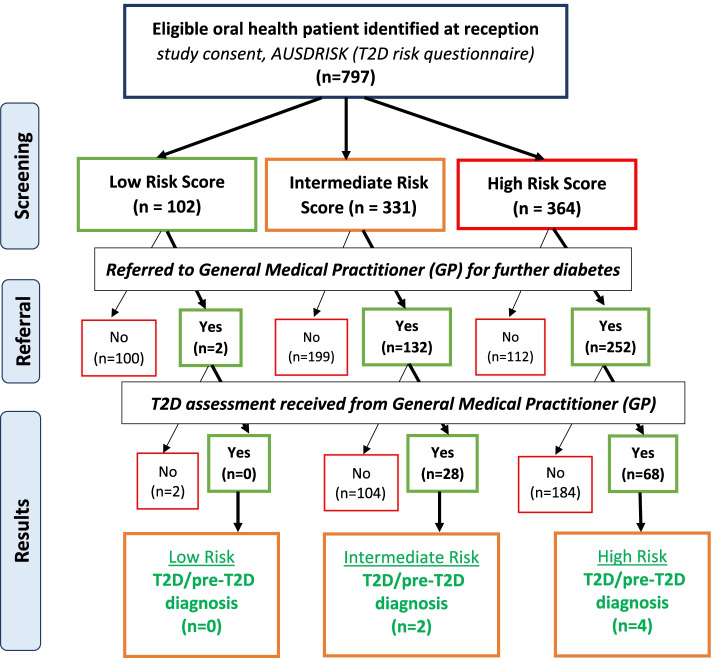
iDENTify patient participant T2D/pre-diabetes screening protocol

One-hundred and two (12.8%) participants were in the low T2D risk group; 331 (41.5%) were in the intermediate T2D risk group; and 364 (45.7%) were in the high T2D risk group. Of the 695 participants who were found to be in either the intermediate or the high T2D risk groups, 384 (55.5%) were referred to their GP for further T2D assessment. Of the referred patients, two was in the low-risk group of having T2D, 132 were in the intermediate risk for T2D group and 252 were in the high risk of T2D group. Two participants in the low-risk category were referred to their GP, due their periodontal condition (See Fig. 2).

A total of 411 participants were not referred to their GP for further T2D assessment. Of these, 100 were in the low risk T2D category, 199 were in the intermediate risk group and 112 were in the high-risk group. Thus, 311 (44.7%) participants that had an intermediate or high AUSDRISK score and should have been referred to their GP for further T2D assessment in accordance with the study protocol, were not referred. The reasons recorded by OHPs for not referring a patient for medical follow-up are included in Table 2. The most frequent reasons included: participants had ‘*good oral health*’ (*n* = 52); OHP did not think the participant had the risk factors (i.e., ‘*low modifiable risk factors*’) that contributed to having T2D or pre-T2D (*n* = 32), another 29 participants informed their OHP they ‘*see their GP regularly for check ups*’ therefore OHP and the participant did not think it was necessary to provide a referral. For nineteen patients the OHP thought the patient had a healthy lifestyle. Fourteen participants advised their OHP they had had a recent T2D screening test and did not have T2D or pre-T2D. Eighteen participants declined a referral without further explanation. There were 148 participants where the OHP did not record a reason for non-referral.

**Table 2:** Reasons for not referring an oral health patient participant to their GP for further pre-type-2 diabetes or type-2 diabetes assessment

		**Decision Maker**
**Not referred to GP**	**411**	
No reason provided by OHP	148	
AUSDRISK Score—Low Risk	98	OHP
Good oral health	52	OHP
Low modifiable risk factors	32	OHP
Patient sees GP regularly	29	Patient / OHP
Patient is fit & healthy, healthy lifestyle	19	OHP
Patient declined GP referral	18	Patient
Patient advised of recent diabetes check	14	Patient
Patient cannot afford a GP appointment	1	Patient

Of the 386 participants referred to their GP for follow-up assessment, a total of 96 results were returned to OHPs; 28 from the intermediate risk T2D score group and 68 from the high risk T2D score group. Of the referred participants, 290 did not attend their GP for further T2D assessment, comprising two from the low risk T2D score group, 104 from the intermediate risk T2D score group and 184 from the high risk T2D score group. Of them, 238 participants did not provide a response or were unable to be contacted (See Table 3). Another 36 declined to see GP. Sixteen participants provided reasons for no T2D assessment results being received, including eight participants who said they saw their GP, but no result was received at the OHP end.

**Table 3:** Reasons provided by the patients for no GP type-2 diabetes assessment results

	**TOTAL**
Total Participants Referred to GP—No Type-2 Diabetes Assessment Result Received	**290**
Declined to see GP	36
Personal issues / other health concerns	2
Cannot afford to see GP	3
No response / unable to contact participant	238
Moved Overseas	1
Participant said they saw their GP – no result received from GP or Participant	8
COVID-19	2

Of the referred participants who did attend their GP for a T2D assessment, and whose results were obtained by the OHP, six (6.3%) were diagnosed with prediabetes two participants from the intermediate risk T2D score group and four from the high risk T2D score group.

## Discussion

A total of 51 private oral healthcare practices and 76 OHPs in both metropolitan and rural Victoria participated in our study. Using the AUSDRISK tool, 87.3% of individuals screened in were identified as being in the intermediate or high T2D risk group. However, nearly half of them were not referred by their OHP for medical follow-up. Additionally, once offered a referral, many participants either declined to or did not attend their GP, Furthermore, some GPs did not communicate the results of their T2D assessment to the referring OHP. As a consequence, only 24.8% of results were received by OHPs. This limits considerably the effectiveness of diabetes screenings. In other studies the percentage of patients attending GPs following a diabetes risk assessment in the oral healthcare setting (private, public and University clinic) has varied from 20 to 84% [4, 19, 27, 33]. In each of these studies patients had consented to undertake screening and had been advised of the possibility of being referred to a GP for follow-up and yet subsequent follow-up was low.

There is evidence that with intensive lifestyle interventions, the progression to T2D may be reduced over time [34]. Still, despite the benefits of lifestyle modification, most individuals with prediabetes are unaware of their condition [35]. Screening for diabetes in the oral healthcare setting therefore may not only aid in the early detection of asymptomatic individuals, but also raise awareness of diabetes risk, and facilitate engagement of at-risk individuals in diabetes prevention. Studies have reported that prediabetes-aware adults were more likely to participate in lifestyle modification than those unaware of their condition [36]. Thus, the results from this study are of potential public health importance, as other patients that undertook the T2D risk assessment, may have benefitted from raising their awareness of prediabetes and T2D, and the modifiable risk factors for developing these conditions.

Barriers to patients attending follow-up appointments with GPs included misplacing the referral letter, being too busy, being away, the perception the condition was not ‘serious enough’ GP’s [27] lack of knowledge about diabetes, not understanding the importance of follow-up, cost, fear and denial [37]. Patients not seeking a medical diagnosis was a barrier to the completion of our screening protocol, and further research is needed to understand the reasons for this and develop strategies to overcome it. Additionally, towards the end of the data collection of the present study, the COVID 19 pandemic impacted the Australian state of Victoria, and may have resulted in some patients not attending medical follow-up. On the other hand, enablers to attending the GP for follow-up have included whether the patient perceived the screening as an opportunity to act, appreciated the ease with which findings could be shared with their GP, perceived empathy from the research team, motivation from observing family members experiencing diabetes or the desire to act as a role model for other family members [37].

Successful implementation of our diabetes screening programme also requires the support of the OHPs who deliver the protocol. In this study, only 55% of those patients identified in the intermediate or the high T2D risk groups were referred to their GP for assessment. In other words, in just under half of individuals screened, the OHP did not follow the protocol guidelines. This represents a potential missed opportunity in identifying an asymptomatic individual with diabetes and may result from deficits in knowledge regarding diabetes risk factors that were highlighted in the Stage 1 of this study [38].

The reasons as to why the intermediate or high-risk patients were not referred by OHPs were investigated. Consistent with the literature, several barriers to OHPs implementing diabetes screening in an oral healthcare setting were cited, including the extra time needed for screening, disruption of the normal running of the oral healthcare practice [27], lack of remuneration [4] and whether the protocol involved an invasive component, such as drawing blood to record capillary blood glucose [37, 39]. Concerns regarding renumeration for diabetes screening, and whether it was within OHP’s scope of practice were cited by OHP’s in the Stage 1 survey but were not raised in the feedback received by OHP’s who participated in the iDENTify screening protocol [38].

Our screening protocol represents a series of steps, beginning with the initial patient engagement and concluding with the OHP receiving a medical diagnosis of the patients they referred to their GP. For the protocol to be effective, co-operation is necessary between OHPs and GPs. OHPs and GPs in Australia deliver care in a fragmented and separated healthcare system. International studies have revealed healthcare provider frustration with the referral process between GPs and OHPs, concluding that improvements are required in the pathways between oral healthcare and general healthcare [40]. A lack of standardised communication channels between OHPs and GPs has been suggested as one reason that levels of follow-up between GP and OHPs have been low in other diabetes screening programmes [41]. For instance, a US study that explored diabetes screening in a student dental clinic initially found none of the 20 GPs responded to the written requests for information on additional diabetes testing results, and only after telephone requests did all GPs provide the test results [42].

In the present study, the return rate of results from GPs to OHPs may have been enhanced by the provision of a referral pack provided to patients to give to their GP. Therefore, the GPs’ knowledge, attitudes and opinions regarding medical screening in the oral health care setting, and receiving referrals from OHPs, may also influence their management of referred patients, and ultimately the effectiveness of the screening protocol. Unlike patients and OHPs, GPs were not invited to provide feedback. Informal feedback from participating GPs in this study, indicated that they believed it was beneficial for OHPs to assess diabetes risk amongst oral healthcare patients and refer these patients to their GP for further investigation. This corresponds to the results in a recent US study, which found most GPs perceived screening for diabetes by OHPs to be valuable, were willing to accept referrals from a dentist, and were willing to communicate with the referring dentist. However, in this US study, two patients referred by their OHP following a diabetes risk assessment, received a refusal from their GPs to perform screening [4]. Thus, highlighting the importance of co-operation between healthcare professionals for diabetes screening to be effective.

This study demonstrates that identifying individuals at an elevated risk of having or developing T2D is effective and feasible in a private oral healthcare setting. Furthermore, our screening protocol reflects an approach that can be easily incorporated into routine private oral healthcare practice. However, for the successful implementation of a screening programme, several barriers need to be addressed, including OHPs’ knowledge deficits regarding diabetes risk factors, and the appropriate management of positive screened cases to ensure the protocol is successful [38]. To maximise the effectiveness of this approach to diabetes screening, improved communication and collaboration is essential between oral health professionals and GPs, to ensure individuals at risk are identified enabling optimal management of their oral health and general health.

## Data Availability

The datasets generated and/or analysed during the current study are not publicly available due to the ethics approval granted on the basis that only researchers involved in the study could access the de-identified data. The minimum retention period is 5 years from publication. Supporting documents are available upon request to the corresponding author.

## Abbreviations

CPD: Continuing Professional Development
T2D: Type 2 diabetes
OHP: Oral healthcare professionals
GP: General Practitioner
HbA1c: Haemoglobin A1c
COVID 19: Corona virus 19
AUSDRISK: Australian Type 2 Diabetes Risk Assessment Tool
DHAA: Dental Hygienists Association of Australia Ltd
ADOHTA: Australian Dental and Oral Health Therapists Association

## References

[1] International Diabetes Federation. Diabetes Atlas (10th ed). International Diabetes Federation. 2021. [cited 2022 February 7]. Available from: https://idf.org/e-library/epidemiology-research/diabetes-atlas.html.

[2] Baker IDI Heart and Diabetes Institute. Diabetes: the silent pandemic and its impact on Australia. [cited 2022 July 15]. Available from: https://baker.edu.au/-/media/documents/impact/diabetes-the-silent-pandemic.pdf?la=en.

[3] Australian Institute of Health and Welfare. Australia’s health 2014. Australia’s health series no. 14. Cat. no. AUS 178. 2014, AIHW: Canberra.[cited 2021 February 7]. Available from: https://www.aihw.gov.au/getmedia/d2946c3e-9b94-413c-898c-aa5219903b8c/16507.pdf.aspx?inline=true.

[4] Genco RJ, Schifferle RE, Dunford RG, Falkner KL, Hsu WC, Balukjian J (2014). Screening for diabetes mellitus in dental practices: A field trial. J Am Dent Assoc.

[5] Diabetes Australia. Type 2 diabetes. 2020. [Cited 2021 February 7]. Available from: https://www.diabetesaustralia.com.au/about-diabetes/type-2-diabetes/.

[6] National Institute for Health and Care Excellence. Preventing type 2 diabetes: risk identification and interventions for individuals at high risk. 2012. NICE Public Health Guidance 38. [cited 2021 February 7]. Available from: https://www.nice.org.uk/guidance/ph38.

[7] Grossi SG, Genco RJ (1998). Periodontal disease and diabetes mellitus: a two-way relationship. Ann Periodontol.

[8] Taylor GW (2001). Bidirectional interrelationships between diabetes and periodontal diseases: an epidemiologic perspective. Ann Periodontol.

[9] Mealey BL (2006). Periodontal disease and diabetes. A two-way street. J Am Dent Assoc.

[10] Fisher MA, Taylor GW, West BT, McCarthy ET (2011). Bidirectional relationship between chronic kidney and periodontal disease: a study using structural equation modeling. Kidney Int.

[11] Soskolne WA, Klinger A (2001). The relationship between periodontal diseases and diabetes: an overview. Ann Periodontol.

[12] Mealey BL, Ocampo GL (2000). Diabetes mellitus and periodontal disease. Periodontol.

[13] Taylor GW, Borgnakke WS (2008). Periodontal disease: associations with diabetes, glycemic control and complications. Oral Dis.

[14] Australian Research Centre for Population Oral Health (2008). The relationship between diabetes and oral health among Australian adults. Aust Dent J.

[15] West B, Parikh P, Arniella G, Horowitz CR (2010). Observations and recommendations for community-based diabetes screenings. Diabetes Educ.

[16] Greenberg BL, Kantor ML, Jiang SS, Glick M (2012). Patients' attitudes toward screening for medical conditions in a dental setting. J Public Health Dent.

[17] Barasch A, Gilbert GH, Spurlock N, Funkhouser E, Persson LL, Safford MM (2013). Random plasma glucose values measured in community dental practices: findings from The Dental Practice-Based Research Network. Clin Oral Investig.

[18] Lalla E, Kunzel C, Burkette S, Cheng BB, Lamster IB (2011). Identification of unrecognized diabetes and pre-diabetes in a dental setting. J Dent Res.

[19] Franck SD, Stolberg RL, Bilich LA, Payne LE (2014). Point-of-care HbA1c screening predicts diabetic status of dental patients. J Dent Hyg.

[20] Grigoriadis A, Räisänen IT, Pärnänen P, Tervahartiala T, Sorsa T, Sakellari D (2021). Prediabetes/diabetes screening strategy at the periodontal clinic. Clin Exp Dent Res.

[21] AlGhamdi AST, Bukhari SMN, Elias WY, Merdad K, Sonbul H (2013). Dental clinics as potent sources for screening undiagnosed diabetes and prediabetes. Am J Med Sci.

[22] Carmagnola D, Demarosi F, Lodi G (2012). Screening for theundiagnosed diabetes at dental chair-side of an Italian univer-sity clinic A pilot prospective study. Minerva Stomatol.

[23] Holm NC, Belstrom D, Ostergaard JA (2016). Identification ofindividuals with undiagnosed diabetes and pre-diabetes in a Danish cohort attending dental treatment. J Periodontol.

[24] Opeodu OI, Adeyemi BF (2013). Undiagnosed diabetes mellitus: a sur-vey of dental outpatients in a tertiary hospital. Afr J Med MedSci.

[25] Barasch A, Safford MM, Qvist V, Palmore R, Gesko D, Gilbert GH, Dental Practice-Based Research Network Collaborative Group (2012). Random blood glucose testing in dental practice: a community-based feasibility study from The Dental Practice-Based Research Network. J Am Dent Assoc.

[26] Wright D, Muirhead V, Weston-Price S, Fortune F (2014). Type 2 diabetes risk screening in dental practice settings: a pilot study. Br Dent J.

[27] Mariño R, Priede A, King M, Adams GG, Lopez D (2021). Attitudes and opinions of Oral healthcare professionals on screening for Type-2 diabetes. BMC Health Serv Res.

[28] Australian Government Department of Health and Ageing. The Australian Type 2 Diabetes Risk Assessment Tool (AUSDRISK). 2020. [cited 2021 February 7] Available from: https://www.health.gov.au/resources/apps-and-tools/the-australian-type-2-diabetes-risk-assessment-tool-ausdrisk/.

[29] Chen L, Magliano DJ, Balkau B (2010). AUSDRISK:an Australian Type 2 Diabetes Risk Assessment Tool based on demographic, lifestyle and simple anthropometric measures. Med J Aust.

[30] Malo JA, Versace VL, Janus ED, Laatikainen T, Peltonen M, Vartiainen E, Coates MJ, Dunbar JA (2015). Evaluation of AUSDRISK as a screening tool for lifestyle modification programs: international implications for policy and cost-effectiveness. BMJ Open Diabetes Res Care.

[31] The Royal Australian College of General Practitioners (2020). Management of type 2 diabetes: A handbook for general practice.

[32] Lwanga SK, Lemeshow S. Sample size determination in health studies: a practical manual. Geneva: World Health Organization; 1991.

[33] Ziebolz D, Bauwe I, Schmidt J, Kottmann T, Rinke S, Schmalz G (2019). Diabetes Screening in Dental Practice Using the Find-Risk Questionnaire - A Practice-based Pilot Study. Oral Health Prev Dent.

[34] Morris DH, Khunti K, Achana F, Srinivasan B, Gray LJ, Davies MJ, Webb D (2013). Progression rates from HbA1c 6.0–6.4% and other prediabetes definitions to type 2 diabetes: a meta-analysis. Diabetologia.

[35] World Health Organization (2016). Global report on diabetes.

[36] International Diabetes Federation (2017). Recommendations For Managing Type 2 Diabetes In Primary Care.

[37] Rosedale MT, Strauss SM (2012). Diabetes screening at the periodontal visit: patient and provider experiences with two screening approaches. Int J Dent Hyg.

[38] Mariño RJ, Adams G, Chinnasamy A, Siva V, King M, Darby I, Morgan M, Temple-Smith M, Furler J, Quinn M, Lau P (2018). Victorian oral health professionals’ knowledge of Type-2 Diabetes. J Dent Res.

[39] Forbes K, Thomson WM, Kunzel C, Lalla E, Lamster IB (2008). Management of patients with diabetes by general dentists in New Zealand. J Periodontol.

[40] Miloro MBM (2016). Vujicic, Physicians dissatisfied with current referral process to dentists.

[41] Glurich I, Bartkowiak B, Berg RL, Acharya A (2018). Screening for dysglycaemia in dental primary care practice settings: systematic review of the evidence. Int Dent J.

[42] Biethman RK, Pandarakalam C, Garcia MN, Whitener S, Hildebolt CF (2017). Screening for Diabetes in a Dental School Clinic to Assess Interprofessional Communication Between Physicians and Dental Students. J Dent Educ.

